# Molecular characteristics and serodiagnostic potential of chitinase-like protein from *Sarcoptes scabiei*

**DOI:** 10.18632/oncotarget.21056

**Published:** 2017-09-19

**Authors:** Ran He, Nengxing Shen, Haojie Zhang, Yongjun Ren, Manli He, Jing Xu, Cheng Guo, Yue Xie, Xiaobin Gu, Weimin Lai, Xuerong Peng, Guangyou Yang

**Affiliations:** ^1^ Department of Parasitology, College of Veterinary Medicine, Sichuan Agricultural University, Wenjiang, China; ^2^ Sichuan Animal Sciences Academy, Sichuan Chengdu, China; ^3^ Department of Chemistry, College of Life and Basic Science, Sichuan Agricultural University, Wenjiang, China

**Keywords:** chitinase-like protein, Sarcoptes scabiei, enzyme-linked immunosorbent assay, immunolocalization, intradermal skin test

## Abstract

Scabies, caused by the mite *Sarcoptes scabiei*, is an allergic skin disease that affects millions of people and other mammals worldwide. This highly contagious parasitic disease is among the top 50 epidemic disease and is regarded as a neglected tropical disease. Diagnosis of scabies is difficult in the early stage, and the pathogenesis of scabies is not currently clear. Here, we expressed, identified and located the chitinase-like protein of *S. scabiei* (SsCLP), and evaluated its potential as an early-stage diagnostic antigen for rabbit scabies. Indirect ELISA using recombinant SsCLP (rSsCLP) exhibited diagnostic sensitivity of 94.4% (17/18) and specificity of 86.7% (26/30). Early diagnostic test after artificial infection of rabbits with *S. scabiei* for 1 week showed a positive detection rate of 96.7% (29/30). Immunolocalization assays showed that fluorescence signals were localized on the surface of mites and, in infected rabbits, were observed in keratinized skin and embedded mites. Intradermal skin tests of rabbits by injecting rSsCLP showed a wheal, flare and erythema reaction. These results suggest that *S. scabiei* chitinase-like protein is conducive to host invasion, participates in inducing the allergic response of the host, and is an effective antigen for the diagnosis of *S. scabiei*.

## INTRODUCTION

Scabies, caused by the mite *Sarcoptes scabiei*, is a parasitic and contagious skin disease that affects humans and other mammals worldwide [1]. Scabies is listed as a neglected tropical disease and represents a significant public health threat, especially in economically disadvantaged populations [2, 3]. It also infects more than 100 species of livestock and wild animals, which results in a significant economic burden [4]. *S. scabiei* mites parasitize the host throughout their life-cycle (adults, nymphs and larvae), and mites can sense the temperature and smell of a host [5]. The pathogenicity of the larvae is the strongest, and direct contact with the skin can cause infection of a healthy host in less than 20 min. The mites burrow into the skin and obtain nutrients from serum and epidermal cells, causing severe itching, scabs and skin thickening, which potentially result in appetite loss, and secondary bacterial infections [6–9].

Chitinase-like protein (CLP) belongs to the glycoside hydrolase family 18 [10], CLPs participate in a variety of biological functions [11–15], including inflammatory reactions and infections. CLPs cleave the chitin of arthropods, fungi and parasites. Some CLPs have no enzymatic activity because of loss of function mutations but they can contribute to airway pathology [16]. It has been reported that CLPs have a critical role in both innate and adaptive type 2 immune responses [17–19], which indicates that they may have the potential for specific antibody detection. Currently, the diagnosis of scabies is mainly based on clinical characteristics, which makes misdiagnosis (e.g., as atopic dermatitis, eczema, hairless tinea or insect bites) a distinct possibility [20]. Thus, searching for an antigenic component of scabies with high diagnostic sensitivity and specificity is a crucial task. Meanwhile, in our transcriptome-microRNA analysis of *Sarcoptes scabiei* and host immune response [21], differential expression analysis showed that CLP of *S. scabiei* was upregulated after host infection, indicating that *S. scabiei* secretes more CLP after the invasion of host. Moreover, the Kyoto Encyclopedia of Genes and Genomes pathway analysis showed that CLP of *S. scabiei* participates in chitosan biosynthesis, chitosan participate in the improvement of immune response [22]. Therefore, exploration of the distribution of CLPs in mites and infected skin may contribute to understanding mite pathogenesis and the immune responses of hosts.

This study aimed to analyze the molecular characteristics of the chitinase-like protein from *S. scabiei*, and explore the role of the CLP in mite pathogenesis. We also assess the serodiagnostic potential of *S. scabiei* CLP.

## RESULTS

### Molecular characterization of *S. scabiei* CLP (SsCLP)

**Figure 1:**
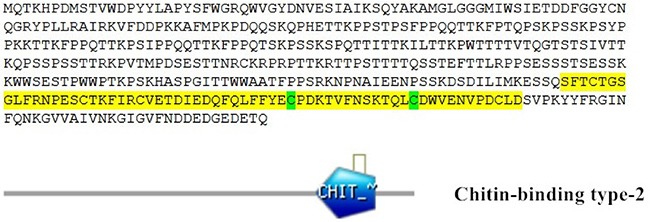
Sequence and functional domain analysis of *Sarcoptes scabiei* chitinase-like protein (SsCLP)

The SsCLP cDNA (GeneBank accession number: KY904739) contained a 1275-bp ORF that encoded a putative protein of 424 amino acids. The predicted molecular mass of the protein was 47.6 kDa, and the isoelectric point was 9.16. No signal peptides were predicted. Multiple sequence alignment revealed that the SsCLP amino acid sequence shares 51–67% overall identity with proteins from *Limulus polyphemus*, *Parasteatoda tepidariorum*, *Tetranychus cinnabarinus*, *Monochamus alternatus*, *Amblyomma sculptum*, *Stegodyphus mimosarum*, and *Megachile rotundata*. The SsCLP amino acid sequence contains a chitin-binding type-2 domain, characterized by a six-cysteine motif and several aromatic residues [23, 24] (Figure 1).

### Expression and identification of rSsCLP

**Figure 2:**
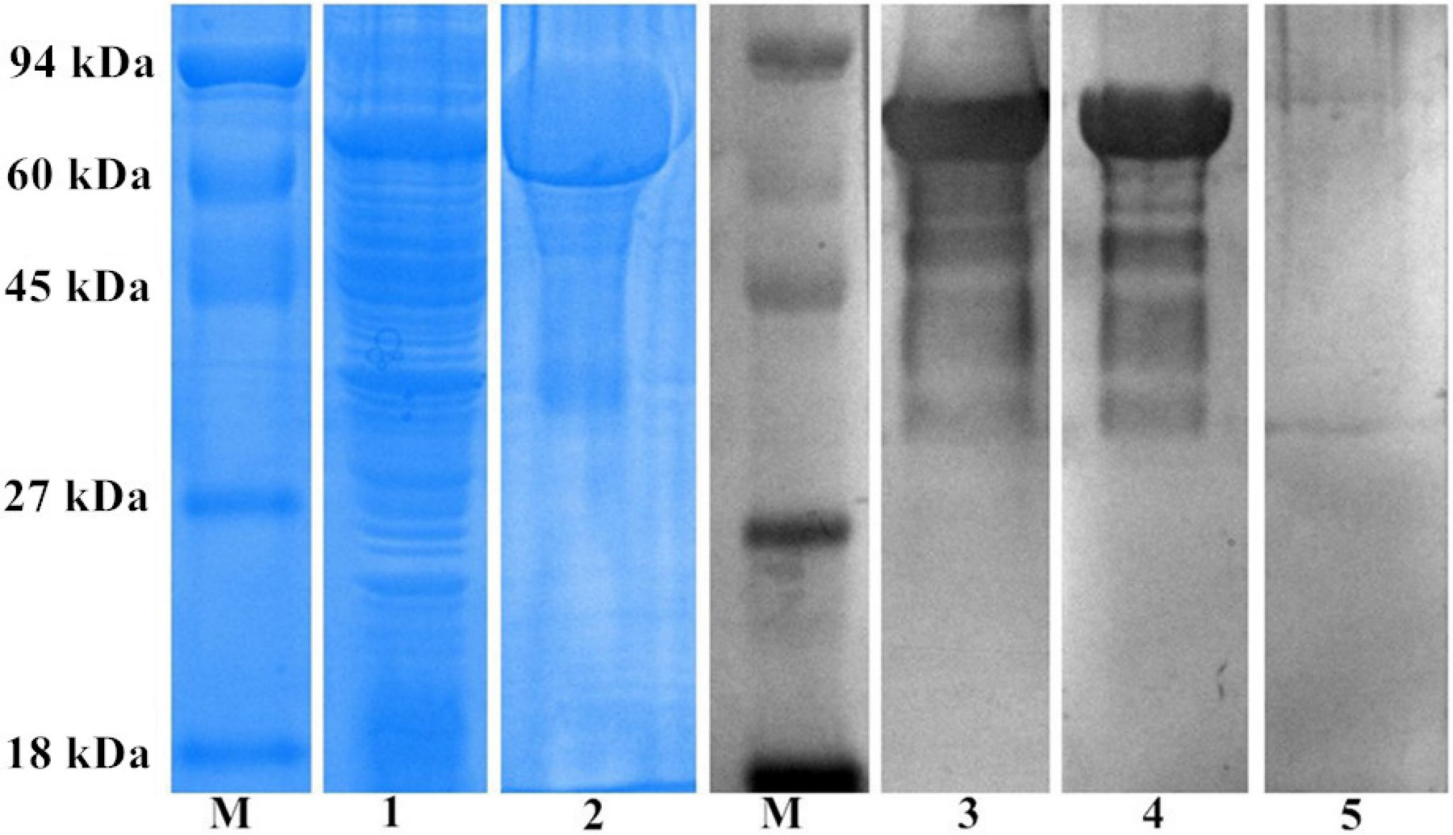
SDS-PAGE and western blotting analysis of SsCLP M, protein molecular-weight markers; lane 1, expressed rSsCLP; lane 2, purified rSsCLP; lane 3, purified rSsCLP probed with anti-rSsCLP rabbit serum; lane 4, purified rSsCLP probed with *S.scbiei* positive rabbit serum; lane5, purified rSsCLP probed with native rabbit serum.

The cDNA encoding SsCLP was successfully sub-cloned into the pET32a (+) expression vector and expressed in *Escherichia coli* BL21 (DE3). The molecular weight (Mw) of recombinant SsCLP (rSsCLP) of approximately 63 kDa (including the His tag) (Figure 2) was similar to the predicted Mw. rSsCLP was purified using a Ni-chelating column and examined by SDS-PAGE (Figure 2). Immunoblotting using anti-rSsCLP rabbit serum and *S. scabiei-*positive rabbit serum showed a single band at approximately 63 kDa, indicating good antigenicity of rSsCLP. No signal was present when rSsCLP was incubated with naïve (healthy) rabbit serum (Figure 2).

### Establishment of indirect ELISA (iELISA)

Using a checkerboard titration protocol, the optimal concentration of the rSsCLP antigen and optimal working dilution of the serum for ELISA were found to be 0.5 μg/well and 1:100 respectively. In these optimized conditions, sera from 12 negative rabbits were analyzed. The optical density was read at 450 nm (OD_450 nm_) using microplate reader (Termo Scientifc, Pittsburgh, PA, USA). The mean OD_450 nm_ value was 0.242 and the standard deviation was 0.0677; thus the cut-off value was calculated as 0.445 (mean + 3SD). The interassay coefficients of variation (CVs) ranged from 1.24% to 5.17% (mean = 3.205%), while the intra-assay CVs ranged from 2.51% to 9.18% (mean = 5.845%). The coefficients were < 10%, which means this assay was repeatable and reproducible.

### Sensitivity and specificity of the iELISA

**Figure 3:**
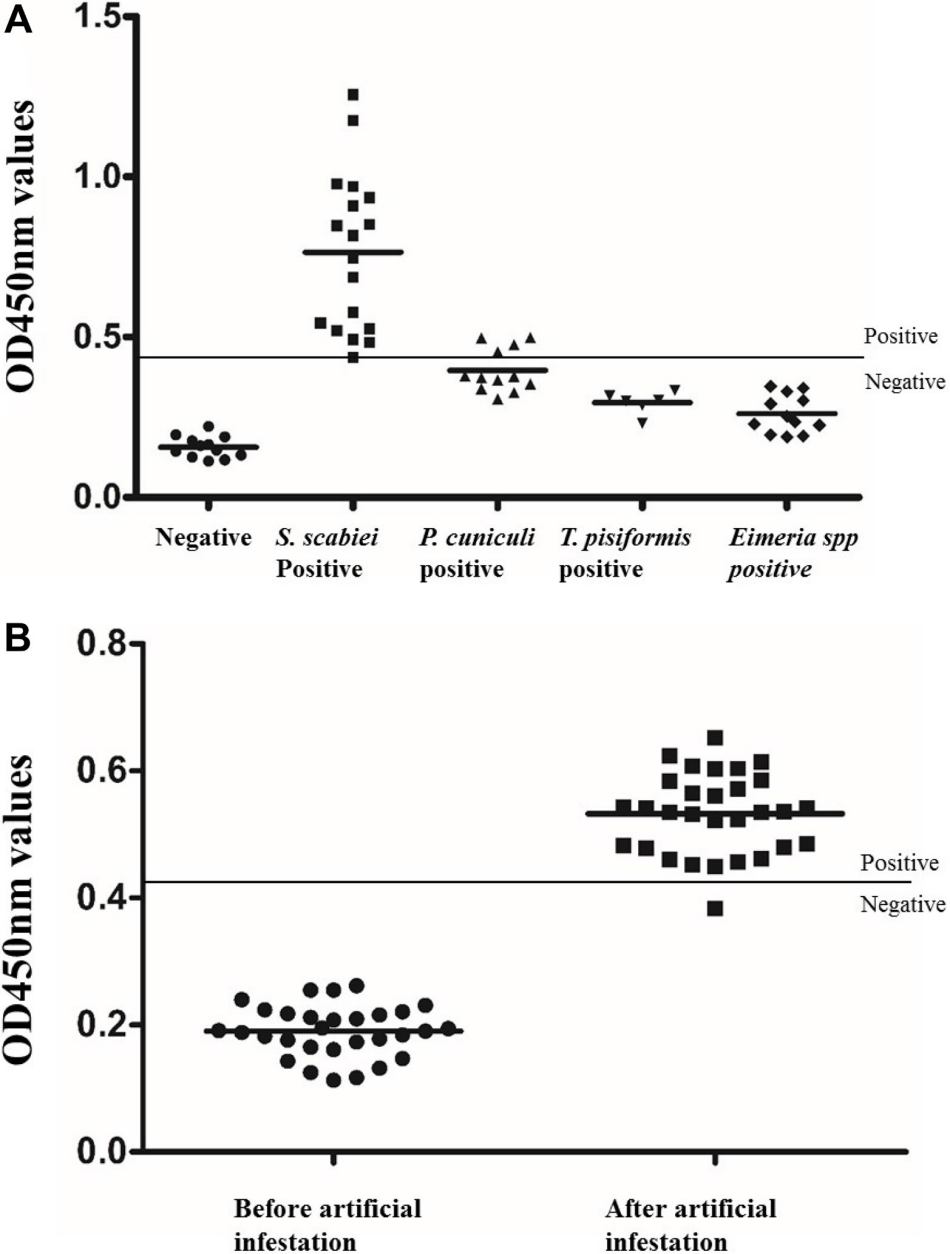
Sensitivity, specificity and early diagnosis test of indirect ELISA ( *P* < 0.05) (**A**) “Negative”: OD_450 nm_ values of rabbit naïve serum samples (*n* = 12); “*S.scabiei* positive”: OD_450 nm_ values of rabbit serum positive for *S.scabiei* ; “*P.cuniculi* positive”: OD_450 nm_ values of rabbit serum positive for *Psoroptes cuniculi* (*n* = 12); “*T. pisiformis* positive”: OD_450 nm_ values of rabbit serum positive for *Taenia pisiformis* (*n* = 6); “*E.*spp positive”: OD_450 nm_ values of rabbit serum positive for *Eimeria* spp (*n* = 12); (**B**) “Before artificial infection”: OD_450 nm_ values of rabbit serum samples that collected before artificial infection (*n* = 30). “After artificial infection”: OD_450 nm_ values of rabbit serum samples that collected after artificial infection for 1 week (*n* = 30).

Serum from 48 rabbits (18 *S. scabiei*-positive, six *Taenia pisiformis*-positive, 12 *Psoroptes cuniculi-*positive, and 12 *Eimeria* spp*.-*positive) were tested in the iELISA. Serum from 17 rabbits infected with *S. scabiei* was detected as positive, corresponding to an assay sensitivity of 94.4% (17/18) (Figure 3A). There was no cross-reactivity between the rSsCLP antigen and serum from rabbits infected with *T. pisiformis* or *Eimeria* spp., but cross-reactivity was observed with four samples of serum from rabbits infected with *P. cuniculi*, corresponding to a specificity of 86.7% (26/30) (Figure 3A). Moreover, statistical differences were observed in the ELISA values between the *S. scabiei*-positive sera and other positive or negative sera (*P* < 0.05; data not shown). No differences were noted between the other positive sera and negative sera.

### Early diagnosis test of iELISA

After artificial infection of 30 rabbits with *S. scabiei*, the OD_450 nm_ of serum samples from 29 rabbits was higher than cut-off value after 1 week (Figure 3B) (*P* < 0.05). The serum samples collected before infection showed lower OD_450 nm_ values than the cut-off.

### Hematoxylin and eosin (H&E) and Immunolocalization

**Figure 4:**
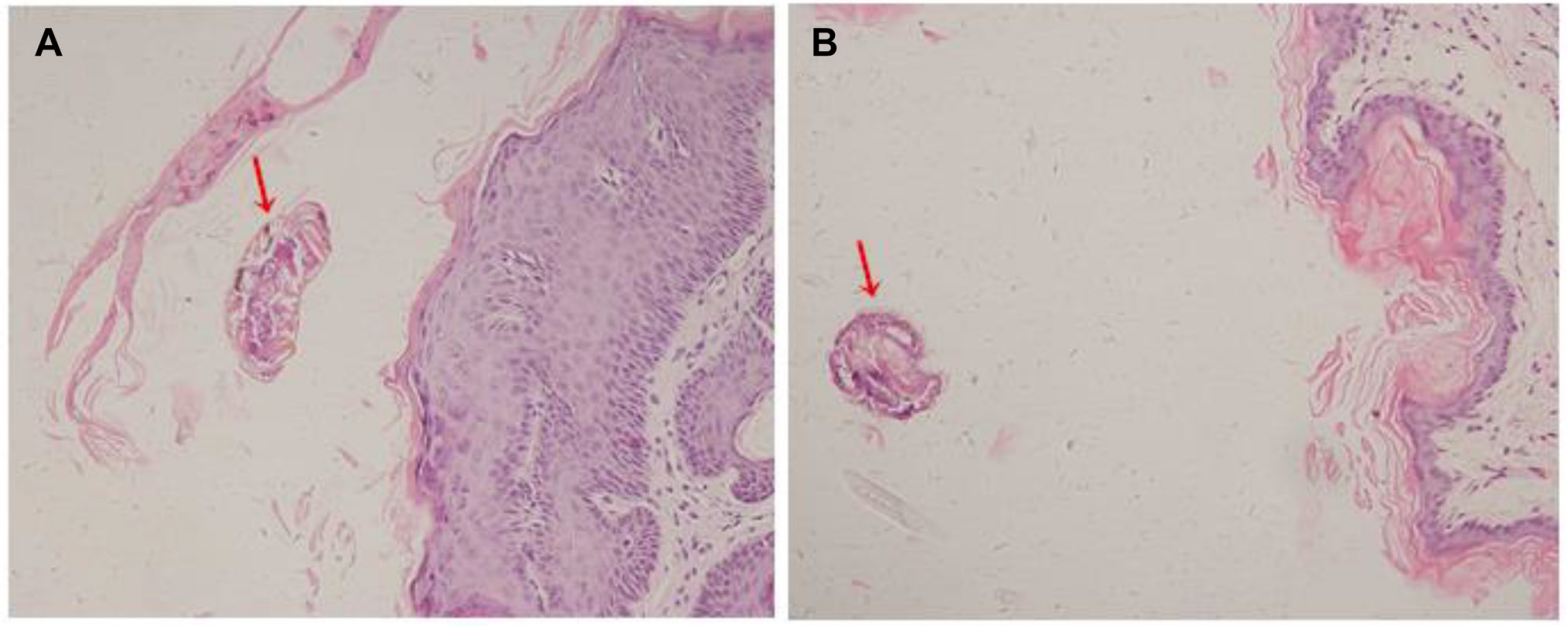
Hematoxylin and eosin (H&E) staining of S.scabiei infected rabbit skin (**A** and **B**). *S. scabiei* invaded the epidermis of skin.

**Figure 5:**
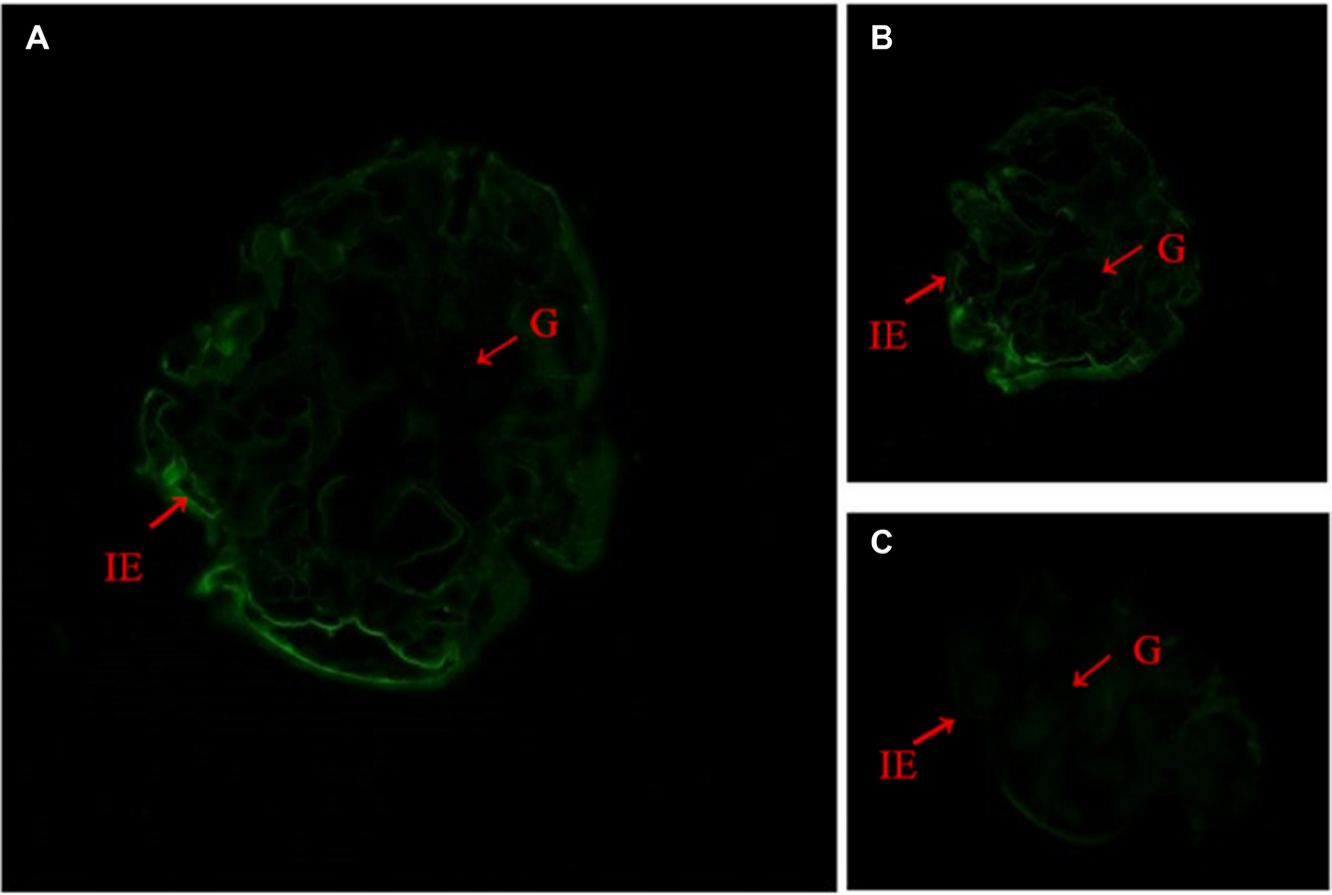
Immunofluorescent localization of SsCLP in starved mites (**A** and **B**), SsCLP was distributed on the surface of mites; (**C**), negative control. Annotation: IE, epidermal integument; G, gut.

**Figure 6:**
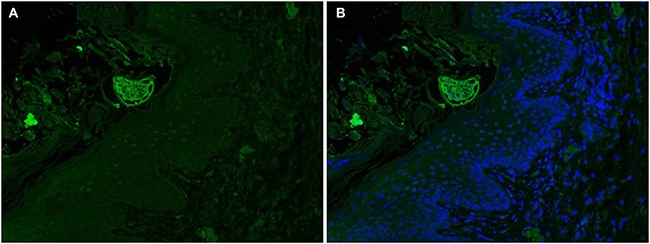
Immunofluorescent localization of SsCLP in *S. scabiei*-infected rabbit skin (**A** and **B**), SsCLP were distributed in the keratinized skin and embedded mite.

H&E staining results showed that scabies invaded the epidermis of the host (rabbit) skin (Figure 4). SsCLP was localized in starved mites and infected skin by an immunofluorescence method. In starved mites, the fluorescence signals were localized on the surface of the mites (Figure 5). In infected rabbits, fluorescence signals were observed in keratinized skin and embedded mites (Figure 6). No fluorescence signals were detected in the negative controls for starved mites (Figure 5) and infected skin.

### Intradermal skin test

**Figure 7:**
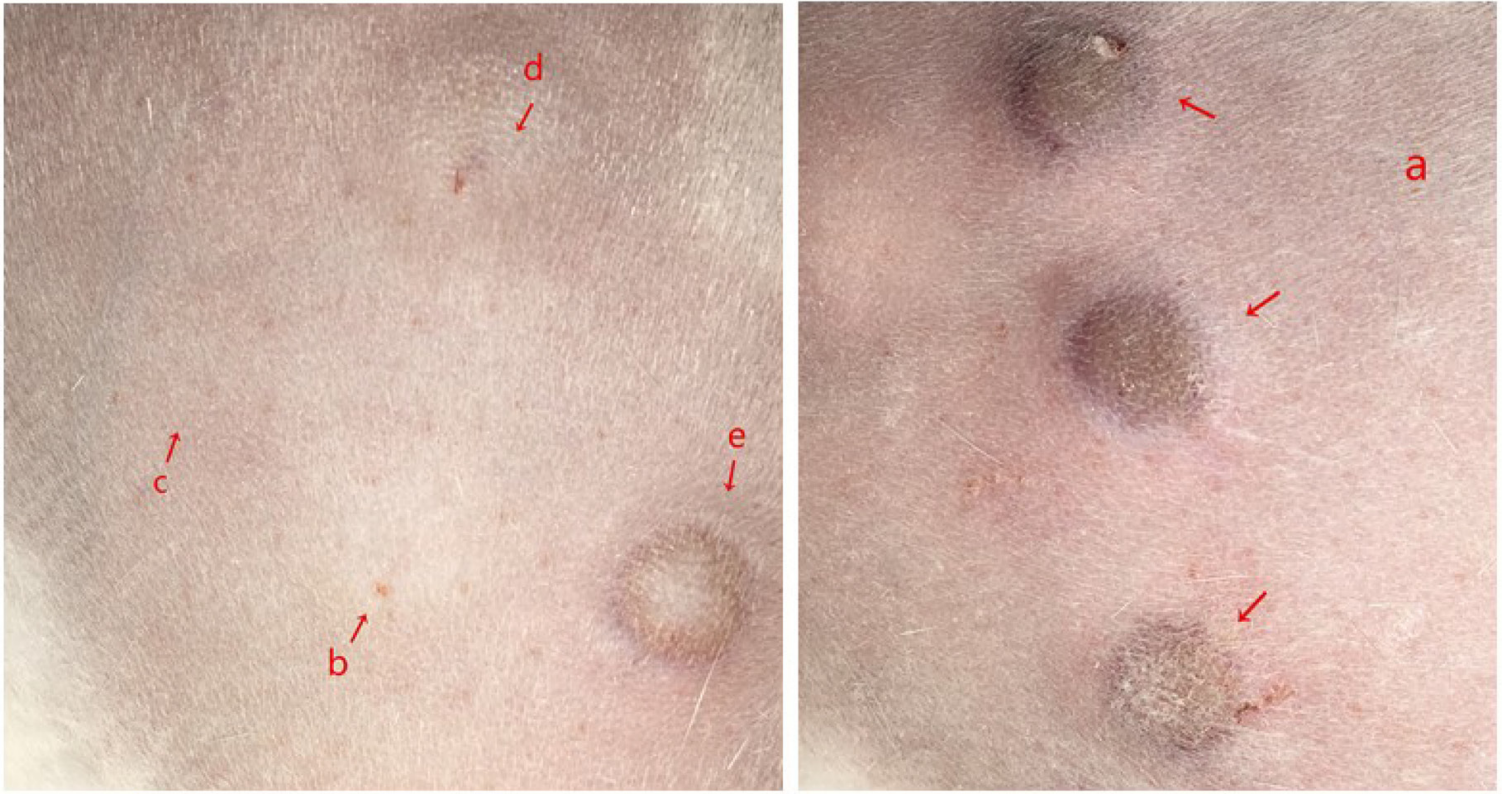
Intradermal skin test on injection of rSsCLP into rabbit a. 400-μg of purified rSsCLP in 0.1ml of PBS; b. 400-μg of purified empty expression vector (pET-32a (+) ) in 0.1ml of PBS; c. 0.1ml PBS; d. physiological saline, 0.9%, 0.1mL; e. histamine, 4mg/mL, 0.1mL.

No reaction was induced by 0.9% physiological saline, PBS or empty expression vector. Histamine, the positive control, produce a wheal reaction. Rabbits injected with rSsCLP showed a wheal, flare and erythema reaction (Figure 7).

## DISCUSSION

Chitinase-like proteins are a mediator family associated with allergy, infection and T cell-mediated inflammation in mammals [25–27]. CLPs were found to limit nematode survival but at the expense of increased lung injury [28]. It has been reported that CLPs are often dysregulated in patients with various disorders and they may influence the Th1 and Th2 balance, affecting inflammatory response and tissue remodeling thus serving as useful diagnostic markers [29, 30]. CLPs have been studied extensively in a variety of organisms, including mammals [19, 31], insects [15] and plants [32, 33], but CLP has not been described in *S. scabiei*. Here we cloned, expressed and established an iELISA method based on rSsCLP to diagnose scabies, and investigated the involvement of SsCLP in the pathogenesis of scabies.

Scabies causes health problems and economic burdens worldwide, and clinical diagnoses depending on clinical signs of *S. scabiei* infection mimic symptoms of other skin diseases [34] (e.g., as atopic dermatitis, eczema, hairless tinea or insect bites). Microscopy diagnoses depending on observation of mites, egg, fecal pellets, eggshell fragments and/or mite fecal pellets from skin scrapings [35], but it is difficult to identify accurately and scraping lesions. Noninvasive diagnoses, such as videodermatoscopy, dermoscopy, reflectance confocal microscopy, and optical coherence tomography, is rapid and no physical risk. But optical coherence tomography and reflectance confocal microscopy are available only in some selected centers. Dermoscopy has been demonstrated more accurate with skin scraping and videodermatoscopy is performed with digital systems (computer and video camera) [36–38]. Mange-detector dogs seem more suitable for wildlife surveillance and control, meanwhile the sensitivity and specificity of this tool has not been identified [39]. Although diagnoses depending on universal conventional and real-time PCR have high specificity and technical sensibility, these diagnosis tools need to collect mites first, and may not suitable for early diagnosis of *S. scabiei* [36, 40–43]. Thus establishing a fast, inexpensive and accurate diagnostic method for *S. scabiei* in live animals is necessary. Here we established an iELISA based on recombinant SsCLP, and it was optimized to diagnose infected rabbits. And rabbit serum positive for *P. cuniculi*, *T. pisiformis*, and *Eimeria* spp. were chosen to perform the cross-reaction, because they are the most common parasites of rabbit.

Previous research using recombinant proteins including Sar s 14.3, thioredoxin peroxidase, Pso o 2, Ssλ20Δ3 and cofilin as antigens in iELISA showed potential value for diagnosis of *S. scabiei* [44–47]. Compared with iELISA using other recombinant proteins as diagnostic antigens, rSsCLP shows considerable sensitivity (94.4%) and specificity (86.7%). In addition, the early diagnosis of 30 rabbit serum samples after artificial infection for only 1 week showed a high positive detection rate of 96.7% (29/30). Interestingly, the OD_450 nm_ values of sera after artificial infection for 2 weeks were not significantly different from those after 1 week. These data indicate that SsCLP participates in the early stage of *S. scabiei*-host immune interaction and induces an early immune response of the host, after which secretion of the host antibody showed no significant change.

H&E staining results showed that scabies invaded the epidermis of the host rabbit skin (Figure 4) *S. scabiei* burrows into the skin and obtains nutrients from the host serum and epidermal cells. Mites dig tunnels and spawn within the skin; larvae can leave the tunnels and drill into skin from the hair follicles [6–8]. In starved mites, CLP fluorescence signals were localized to the surface of the mites (Figure 5A, 5B), and in infected rabbits, fluorescence signals were observed in keratinized skin and embedded mites (Figure 6). Thus, SsCLP is distributed on the surface of the mites and dispersed in the infected skin of the host. It has been reported that secretory proteins play a key role in infection of parasites, as well as in immunoregulation of the host and immune system evasion [48, 49]. Parasites produce secretory proteins and release them directly into host fluids and tissues.

Secretory proteins of some parasites (such as mites) are important allergens that induce an inflammatory response of the host [50–52]. Sixty-one allergen unigenes have been predicted in the transcriptome of *S. scabiei* canis [53], and groups of allergen genes had been identified in the draft genome of the scabies mite [2]. When we compared our transcriptome data with dust mite allergen, we found homologous allergen unigenes. Among genome and transcriptome of *S. scabiei canis* and transcriptome of *S. scabiei* *cuniculi*, CLP share more homology with group 15 allergen of the house dust mite (HDM). The sequence we cloned and expressed contained a chitin-binding type-2 domain. Group 15, 18 and 23 dust mite allergens contain sequences similar to chitin-binding domains, and have been identified as major HDM allergens [54]. These proteins containing chitin-binding domain may provide important evidence for nutrient procurement, digestion and defecation of *S. scabiei* [55]. In intradermal skin tests (Figure 7), rSsCLP injection into rabbit induced a wheal, flare and erythema reaction. Scabies is an allergic skin disease caused by allergens, most mite allergens are proteases and they can promote inflammation and allergic reactions, including rhinitis, asthma and dermatitis [56–58]. It has been reported that Der p 1 and Der p 2 (allergens from the house dust mite *Dermatophagoides* *pteronyssinus*) may increase allergic immune responses of the host by respectively cleaving IgE receptors and boosting the innate immune response [59, 60]. Cockroach allergens induce immunologic responses by activating innate immune cells (such as dendritic cells) by binding to either Toll-like receptors or C-type lectin receptors [61]. These findings suggest that *S. scabiei* allergens may play a crucial role in the pathogenesis of scabies. Wheal, flare and erythema reactions are common symptoms of allergic skin disease, indicating that rSsCLP can induce specific, immediate hypersensitivity responses. Moreover, H&E staining and immunolocalization provided evidence of mite invasion and protein distribution in the skin, and the early diagnosis iELISA indicated that SsCLP participated in the early stage of the *S. scabiei*-host immune interaction and induced an immune response of the host. These results correspond to our transcriptome-microRNA analysis results, and suggest that SsCLP is conducive to host invasion, participates in inducing the allergic response of the host, and is an effective antigen for the diagnosis of *S. scabiei*.

## MATERIALS AND METHODS

### Ethics statement

Animals were handled strictly according to the animal protection law of the People's Republic of China (released on 09/18/2009) and the National Standards for Laboratory Animals in China (executed on 05/1/2002). This study was reviewed and approved by the Animal Ethics Committee of Sichuan Agricultural University (China) (Approval No. 2013–028). All the methods were carried out in accordance with all relevant guidelines and regulations.

### Mites and animals

Mites (adults, nymphs and larvae) collected from rabbits were provided by the Department of Parasitology, College of Veterinary Medicine, Sichuan Agricultural University. The mites were stored in liquid nitrogen prior to RNA extraction and were unfed prior to experiments. New Zealand White rabbits (8-weeks-old) were obtained from the Laboratory Animal Center of Sichuan Agricultural University (Ya’an, China). All rabbits were housed in a barrier environment in sterile cages and provided with pelleted food and sterilized water *ad libitum*. Rabbits were acclimated to these conditions for 1 week before experiments.

### Bioinformatic analysis of SsCLP

The SsCLP sequence was obtained from our transcriptome data of *S.scabiei* (NCBI Bio Project ID: PRJNA320671). Biochemical parameters such as molecular weight and isoelectric point were predicted using the ExPASy Proteomics Server ( http://web.Expasy.org/protparam/). The open reading frame (ORF) was determined using ORF Finder ( http://www.ncbi.nlm.nih.gov/orffinder/). Signal peptides were predicted using the SignalP 4.1 Server (http://www.cbs.dtu.dk/services/SignalP/). Multiple sequence alignment was performed using DNAMAN 3.0 (Lynnon Biosoft, Canada). The amino acid sequences were translated using BioEdit ( http://www.mbio.ncsu.edu/BioEdit/bioedit.html#downloads) and functional domains were scanned using PROSITE ( http://prosite.expasy.org/scanprosite/).

### Expression and identification of rSsCLP

Total RNA was isolated from mites by using an RNA Extraction Kit (Cowin Biotech, China), according to the manufacturer's instructions. cDNA was transcribed using the RevertAi™ First Strand cDNA Synthesis Kit (Thermo, USA), according to the manufacturer's instructions, and stored at −80°C. The region that encodes rSsCLP was amplified for expression by using primers (5′-GGGGTACCTTGTGAAATGCAAACTAA-3′; 5′-CG AGCTCTTCACTGAGTTTCATCCT-3′).

The polymerase chain reaction procedure was: 94°C for 5 min followed by 30 cycles of 94°C for 40 s, 58°C for 45 s, and 72°C for 1 min. The samples were then incubated at 72°C for 10 min and then held at 8°C. The PCR products were digested and gel-purified, cloned into the vector pMD19-T (TaKaRa, China), and then ligated into the BamHI/XhoI restriction sites of vector pET-32a (+) (Novagen, USA). The recombinant plasmid was transfected into *E. coli* BL21 (DE3) cells (Invitrogen, USA), and expression was induced with 1 mM isopropyl β-D-1-thiogalactopyranoside at 37°C for 16 h. The cells were harvested and resuspended in lysis buffer (50 mM NaH2PO4, 10 mM Tris–HCl, pH 8.0), 100 mM NaCl), and ultrasonic lysis was performed. Then, rSsCLP was purified by Ni^2+^ affinity chromatography (Bio-Rad, USA), according the manufacturer's instructions; rSsCLP was detected using 12% SDS-PAGE, and the concentration of rSsCLP was estimated using a Bicinchoninic Acid Protein Assay Kit (Pierce, USA).

### Serum and anti-serum preparation

Negative serum was collected from 12 healthy New Zealand White rabbits, and serum positive for *S. scabiei* (*n* = 18) was collected from naturally infected New Zealand White rabbits in Wenjiang, Sichuan Province, China. Rabbit serum positive for *P. cuniculi* (*n* = 12), *T. pisiformis* (*n* = 6), and *Eimeria* spp. (*n* = 12) was also obtained from Wenjiang. Meanwhile, 30 healthy rabbits were artificially infected with an equal weight of mites (approximately 2000 live mites) on the surface of all four feet wrapped by gauze for 48 h. Serum samples were collected before infection (healthy serum) and after infection for 1 and 2 weeks. All serum samples were stored at −20°C.

For anti-serum preparation, 200 μg rSsCLP emulsified with an equal volume of Saponin adjuvant (Sigma, USA) was injected subcutaneously into New Zealand White rabbits at first immunization. Second and third booster injections were given at 2-week intervals and consisted of 100 μg protein with equal volumes of Saponin adjuvant. The last immunization was an injection of 200 μg rSsCLP. The rabbit antisera were collected 1 week after the final immunization. The antibody titer was determined by ELISA. Immunoglobulin G (IgG) was isolated from the antisera using a Protein G-Sepharose column (Bio-Rad) according the manufacturer's instructions.

After purification, rSsCLP was mixed with electrophoresis sample buffer and boiled for 10 min. Then, the samples were separated by 12% SDS-PAGE and transferred onto nitrocellulose membranes for 35 min in an electrophoretic transfer cell (Bio-Rad). The membrane was blocked with 5% skim milk for 2 h, followed by incubation overnight with IgG or serum positive for *S. scabiei* (1:100 dilution) at 4°C. Then, the membrane was washed three times with Tris-buffered-saline-Tween20 (TBST) and incubated for 2 h with horseradish peroxidase (HRP)-conjugated goat anti-rabbit IgG (1:3000; Boster, China). The protein signals were visualized with the Enhanced HRP-DAB Chromogenic Substrate Kit (Tiangen, China), according to the manufacturer's instructions. The serum of naïve (healthy) rabbits was used as the negative control.

### iELISA development and serum detection

iELISA was used to evaluate the serodiagnostic potential of rSsCLP. It was performed in polystyrene 96-well microtiter plates (Invitrogen) coated overnight at 4° C with 100 μL reaction mixtures of eight different concentrations of rSsCLP in 0.1 M carbonate buffer (pH 9.6). After washing three times with 0.1 M phosphate-buffered saline containing Tween 20 (PBST), each well was blocked with 100 μL of skim milk in PBS (5 %) for 90 min at 37° C. Serial twofold dilutions (in PBS) of positive and negative serum samples were used in the following steps. After washing three times with PBST, goat anti-rabbit IgG-HRP conjugate (100 μL, 1: 3000; Earth Ox, USA) was added to each well and incubated for 60 min at 37°C. Antibody binding was detected with 100 μL of 3,3′,5,5′-tetramethylbenzidine (TMB; Tiangen, China), and the optical density was measured at 450 nm. After the optimal dilutions of rSsCLP antigen and rabbit serum were determined, the cut-off value was defined as the mean OD_450 nm_ absorbance value plus three times the standard deviation from the 12 negative rabbit serum samples.

Intraplate repeatability was evaluated by the coefficient of variation (CV %) of every serum sample. Three separate assays were used to evaluate the interplate repeatability.

### Sensitivity and specificity of indirect ELISA analysis

To evaluate the sensitivity of the rSsCLP iELISA, 18 *S. scabiei*-positive rabbit sera were tested. The specificity was evaluated from the cross-reaction with antibodies derived from rabbit serum positive for *T. pisiformis* (*n* = 6), *P. cuniculi* (*n* = 12), and *Eimeria* spp. (*n* = 12). The sensitivity of the assay was calculated as: sensitivity (%) = ELISA positive/true positive × 100. The specificity of the assay was calculated as: specificity (%) = ELISA negative/true negative × 100.

### Early diagnosis test by iELISA

To assess the early diagnosis potential of rSsCLP, serum samples from 30 rabbits were tested after artificial infection with *S. scabiei* for 1 and 2 weeks. The diagnostic rate was calculated based on the cut-off value. Each serum sample was tested three times.

### H&E staining and fluorescence immunohistochemistry assay

Starved mites (mites collected and starved for 12 h before being stored in liquid nitrogen) and skin samples were collected from the feet of *S. scabiei*-infected rabbits. Samples were fixed in 1% molten agarose and set in paraffin wax after solidification of the molten agarose. The infected skin and starved mite samples were fixed in 4% paraformaldehyde in PBS (pH 7.4) for 24 h. A rotary microtome was then used to cut the embedded samples into 5-μm-thick sections.

To make the results of fluorescent immunohistochemistry clearer, H&E staining was performed as a control. Then the sections were rehydrated by immersing the slides successively in xylene (twice, for 7 min each), 100% ethanol (twice, for 3 min each), 95% ethanol for 3 min, 85% ethanol for 3 min, 75% ethanol for 3 min, and deionized sH_2_O for 8 min followed by blocking with 3% H_2_O_2_. The sections were then washed and incubated overnight with purified IgG fractions (diluted 1:100 in PBS) at 4°C. Subsequently, the sections were washed and incubated with fluorescein isothiocyanate-conjugated goat anti-rabbit IgG (H + L; AMRESCO, Texas, USA) diluted 1:100 in 0.1% Evans blue at 37°C for 1 h in darkness. After washing three times with PBS, the samples were mounted with an anti-fade mounting medium and visualized using a fluorescence microscope. Negative controls were performed with preimmune rabbit serum.

### Intradermal skin test

Because of the lack of an effective secondary anti-rabbit IgE antibody, IgE ELISA and IgE dot blot are unavailable. Therefore, the allergenic activity of rSsCLP was preliminarily investigated by intradermal skin tests. Three New Zealand White rabbits were injected subcutaneously with 400 μg purified rSsCLP in 0.1ml PBS. The negative controls were: (i) 0.1 mL PBS; (ii) physiological saline, 0.9%, 0.1 mL; and (iii) 400 μg of purified empty expression vector (pET-32a (+) ) in 0.1 mL PBS. The positive control was histamine, 4 mg/mL, 0.1 mL. Skin reactions were observed in the following 30 min and defined as positive with the presence of a wheal and erythema.
